# Neuropsychological assessment in the Israeli healthcare system: a practitioners’ survey

**DOI:** 10.1186/s13584-020-00403-3

**Published:** 2020-09-14

**Authors:** Gitit Kavé, Ayala Bloch, Adi Shabi, Sari Maril

**Affiliations:** 1 Department of Education and Psychology, The Open University, 1 University Road, 4353701 Ra’anana, Israel; 2 Center for Memory and Attention Disorders, Sourasky Medical Center, Tel Aviv, Israel; 3 The National Institute for the Rehabilitation of the Brain Inured, Tel Aviv, Israel; 4 Department of Psychology, Ariel University, Ariel, Israel

**Keywords:** Neuropsychological assessment, Neuropsychological evaluation, Cognitive evaluation, Neuropsychological practice, Neuropsychological rehabilitation

## Abstract

**Background:**

The current study examines self-reported professional practices and attitudes of Israeli neuropsychologists, in an attempt to understand how they contribute to funding of neuropsychological assessment (NPA) through the Israeli healthcare system.

**Methods:**

Two hundred seventy-nine neuropsychologists (176 board-certified experts and 103 interns) participated in an online survey that targeted characteristics of NPA practice in Israel, attitudes toward NPA, and familiarity with healthcare referral procedures.

**Results:**

Overall, 68% of respondents conducted NPA, with a smaller proportion of experts (56%) doing so than interns (88%). The most common purpose of NPA was to provide treatment recommendations, and respondents listed indications for NPA that matched indications for neuropsychological rehabilitation. Almost two thirds of respondents reported that none of the NPAs that they performed received healthcare funding. While all practitioners believed that the healthcare system should fund NPA, the majority demonstrated lack of familiarity with referral procedures.

**Conclusions:**

To increase referral rates and create effective neuropsychological services within the Israeli healthcare system, neuropsychologists should work more closely with physicians in integrated care teams. In addition, they should engage in greater advocacy activities that will emphasize the need for publicly funded NPA.

Neuropsychology is a branch of psychology that serves individuals with brain damage due to disease, head trauma, or developmental disorders. Neuropsychological assessment (NPA) can provide valuable information about these individuals [1, 2], and it has the potential to improve diagnostic classification, prediction of disease outcomes, and care [3]. For example, an NPA can indicate whether a concussion resulted in cognitive decline and other psychological difficulties, or help determine whether aging-related changes in cognition are within the normal or the pathological range. Despite these benefits, an inquiry with the two largest Israeli health plans (HP), which serve approximately 6,710,000 members, revealed that they funded only 412 NPAs in 2018. We have recently argued that this pattern of under-referral stems in part from the lack of familiarity with NPA among Israeli physicians [4]. In the present study, we examine the contribution of neuropsychologists to this paucity of healthcare funding.

Israel has a universal National Health Insurance Law that mandates health services to all citizens and permanent residents, regardless of employment status or age. Every person belongs to one of four HPs, and HPs must provide their members with access to a statutory benefit package [5]. This package includes doctor visits, diagnostic and laboratory tests, hospitalizations, subsidized prescription medications, and psychological services. The law covers NPA, reflecting the understanding that NPA is important for accurate diagnosis and for appropriate care.

To be entitled to an HP-funded NPA, a person must receive a referral letter from a relevant expert, and the HP must provide a payment voucher indicating that it agrees to pay for the service. Importantly, the HPs outsource almost all NPA services, and these take place primarily in hospital-based neuropsychological clinics and in some non-profit organizations. The HP pays approximately 1500 NIS for a focused NPA. The National Insurance Institute of Israel and the Rehabilitation Department of the Ministry of Defense also pay for NPA, but they do so outside the healthcare system, primarily through non-profit organizations. In addition, individuals who need NPA can pay for these services privately.

Vakil and Hoofien [6] have previously reported that 75% of Israeli neuropsychologists worked at least part time in a public service, such as hospitals or non-profit organizations. In principle, then, they can provide NPAs through the healthcare system. Yet, the extent to which neuropsychologists focus on assessment in Israel appears to be limited relative to other countries. A survey of the practice characteristics of 512 neuropsychologists in North America showed that practitioners devoted an average of 59% of their professional time to NPAs, performing approximately three NPAs per week [7]. In Argentina, 91% of neuropsychologists reported that they conducted NPA regularly, with an average of 15 NPAs per month [8], and in Spain, 88% of neuropsychologists conducted NPA, with an average of 18 NPAs per month [9].

Compared to neuropsychologists in other countries, Israeli practitioners are more involved in case management, vocational counseling, and rehabilitation psychotherapy [6]. This focus is rooted in the development of the profession in Israel as well as in its current practice. Historically, Israeli neuropsychology evolved from rehabilitation psychology [6], placing greater emphasis on treatment than on assessment. Vakil and Hoofien [6] looked at a sample of 121 Israeli practitioners, 52% of them interns, and found that 77% of Israeli neuropsychologists combined therapy and assessment, 15% focused on therapy alone, and only 8% focused on assessment alone. Moreover, at present, there is no specific accreditation in neuropsychology in Israel, and most practitioners complete internships in Clinical or Rehabilitation Psychology. As of January 2018, the internship in Rehabilitation Psychology requires 240 h of supervision, 60 of which should focus on assessments, as well as completion of 12 full assessments. These requirements assign clear priority to psychotherapy and rehabilitation interventions over assessment. As the previous survey of Israeli neuropsychologists [6] included more interns than experts, and since it did not focus specifically on NPA or on the healthcare system, we set out to examine how practitioners’ practice might affect healthcare funding of NPA.

Previous surveys of practicing neuropsychologists in North and South America as well as in Europe suggest that the most frequently endorsed assessment referral question is the determination of diagnosis [7–11]. These surveys also show that neurologists are the number one referral source for NPA [9, 10], yet NPA referrals also come from psychiatrists and general medical practitioners [7]. In a complementary survey of American physicians, 65% of 517 respondents indicated that the most common reason for patient referral to NPA was to establish or confirm a diagnosis, and 36% indicated that they referred patients in order to receive treatment recommendations [12]. We therefore examine the extent to which Israeli neuropsychologists conduct NPAs that target physicians’ questions and needs.

In summary, the Israeli National Health Insurance Law supports healthcare funding of NPA, and the majority of Israeli neuropsychologists work in public service. Yet, rates of NPA funding within the healthcare system are low. In the present study, we investigate characteristics of practitioners who conduct NPA in Israel, as well as their attitudes toward NPA. Our underlying premise is that several aspects of the field of neuropsychology in Israel could contribute to under-funding of NPA within the Israeli healthcare system.

## Methods

### Participants

We received the number of practitioners in Rehabilitation Psychology from the Israeli Ministry of Health in June 2018. At that time, the records included 368 board-certified experts, 117 of them accredited as supervisors, as well as 293 interns. Due to privacy policies, the Ministry of Health could not provide us with the email addresses of these individuals. However, Israel has an official online registry of psychologists that presents the names of all board-certified experts in psychology, together with their field of expertise (but with no contact information). Thus, we searched the registry for the names of all experts, and then looked for relevant email addresses through mailing lists of previous conferences, professional groups on social media, online institutional phone directories, and personal connections. This search resulted in 318 email addresses of board-certified experts in Rehabilitation Psychology (86% of those listed in the registry), 102 of them supervisors (87% of those listed in the registry), and 54 board-certified experts in other fields of psychology whose practice involved neuropsychology. The search for interns was more complicated, because the registry presents their names without specifying the field in which they train. We used similar sources to obtain names and mailing addresses of 194 registered psychologists who were training in Rehabilitation Psychology (66% of interns). We then sent an email with a link to the survey to 566 potential respondents and invited them to complete an anonymous questionnaire. Recruitment took place between September and November 2018, with one reminder sent in October. The study received Institutional Review Board approval.

The final sample included 279 respondents, representing an overall response rate of 49% relative to survey invitations. Participants reported 1–46 years in practice, suggesting that we recruited individuals with highly diverse experience (see Table 1 for full demographic details). There were 157 board-certified experts in Rehabilitation Psychology (41% of those listed in the registry), and 19 board-certified psychologists with various other types of expertise (e.g., Clinical, Educational, or Medical Psychology). Seventy-four respondents were accredited supervisors (26% of the sample, 42% of board-certified psychologists in the sample), and 66 of them were accredited as supervisors in Rehabilitation Psychology (56% of those listed in the registry). The number of participating interns (*N* = 103) represented a response rate of 53% relative to survey invitations. However, this number amounted to only 35% of interns listed in the registry, reflecting our difficulty in obtaining a complete list of interns. Twenty-three percent of the entire sample held a doctoral degree, and the rest held a Master’s degree. The majority of the sample (75%) worked in central Israel, 15% worked in the northern part of the country, and 10% worked in the south.

**Table 1:** Sample characteristics, by respondents’ career stage

	Experts	Interns	Total
N	176 (63%)	103 (37%)	279
Female (%)	131 (74%)	79 (77%)	210 (75%)
Mean age (SD)	49.05 (10.93)	33.28 (3.45)	42.96 (11.70)
Age range	33–80	25–49	25–80
Mean years in practice (SD)	19.01 (10.58)	4.17 (2.51)	13.52 (11.14)
PhD (%)	57 (32%)	7 (7%)	64 (23%)

Note: Numbers in the top row reflect the percentage of participants relative to the total number of participants. Other numbers reflect the percentage of participants relative to the total number of experts (second column), the total number of interns (third column), and the total number of participants (fourth column)

### Questionnaire

The survey consisted of 50 questions that addressed four main topics: demographic background (e.g., age, sex); characteristics of NPA practice (e.g., source of referrals); attitudes toward NPA (e.g., which type of patients should receive NPA?); and familiarity with referral procedures (e.g., have you heard of codes L9617 and L9616?). Nineteen questions required a binary answer (e.g., do you conduct NPA?). Thirteen questions presented multiple-choice alternatives (e.g., which percent of the NPAs that you conduct is HP-funded?). Seven questions required ratings on a Likert scale (e.g., how difficult is it for your clients to receive HP funding for NPA?). Eleven questions required typing a verbal response (e.g., what are the advantages of NPA over other cognitive evaluations?).

## Results

The survey showed that 72% of the sample worked in public service, with 121 respondents (43%) working in a hospital, 107 respondents (38%) working in a non-hospital public service (e.g., non-profit organizations), and 28 respondents (14 experts and 14 interns) working in both types of services. In addition, the majority of board-certified experts (82%) reported working in a private clinic, and 12% reported working only in public service.

Overall, 68% of respondents reported that they conducted NPA (see Table 2). The proportion of experts who conducted NPA (56%) was significantly lower than the proportion of interns who did so (88%), *X*^*2*^ = 31.175, *p* < .05. Almost a third (31%) of the respondents who did not conduct NPA stated that the reason for not conducting assessments was their professional preference, while others mentioned work place circumstances or lack of appropriate training. Seventy percent of those who conducted NPA saw up to 10 cases per year (see Table 2). The proportion of experts (60%) who saw up to 10 cases per year was lower than the proportion of interns (84%) who did so, *X*^*2*^ = 12.017, *p* < .05. Sixty-one respondents reported that they conducted NPA for court, amounting to 22% of the sample and 32% of those who conducted NPA. When asked to report the purpose of the last NPA that they conducted, 38% of the respondents listed treatment recommendations, 34% mentioned a medico-legal reason, 20% stated differential diagnosis, 3% conducted NPA to establish pre-surgical baseline, and 5% cited other reasons. The majority of both experts and interns reported that physicians and other psychologists were the main source of referrals, followed by the National Insurance Institute of Israel and the Rehabilitation Department of the Ministry of Defense. Of the respondents who conducted NPA, 124 (65%) reported that none of the NPAs that they conducted received HP funding, and experts reported greater lack of HP funding (74%) than did interns (56%), *X*^*2*^ = 7.108, *p* < .05.

**Table 2:** Characteristics of neuropsychological assessment (NPA) practice, by respondents’ career stage

Question	Experts	Interns	Total
Do you work in a hospital? (% yes)	64 (36%)	57 (55%)	121 (43%)
Do you work in a non-hospital public service? (% yes)	66 (38%)	41 (40%)	107 (38%)
Do you conduct NPA? (% yes)	98 (56%)	91 (88%)	189 (68%)
Do you conduct NPA for court? (% yes)	44 (25%)	19 (18%)	61 (22%)
How many NPAs did you conduct in the past year?	*N* = 91	*N* = 75	*N* = 166
1–2	17 (19%)	21 (28%)	38 (23%)
3–5	19 (21%)	27 (36%)	46 (28%)
6–10	18 (20%)	15 (20%)	33 (20%)
11–19	16 (17%)	6 (8%)	22 (13%)
20+	21 (23%)	6 (8%)	27 (16%)
What was the purpose of last NPA that you conducted?	*N* = 93	*N* = 85	*N* = 178
Treatment recommendations	30 (32%)	38 (45%)	68 (38%)
Medico-legal	34 (37%)	27 (32%)	61 (34%)
Differential diagnosis	22 (24%)	13 (15%)	35 (20%)
Pre-surgical baseline	1 (1%)	4 (5%)	5 (3%)
Other	6 (6%)	3 (3%)	9 (5%)
Who refers your clients to NPA?	*N* = 95	*N* = 80	*N* = 175
Physicians	69 (73%)	56 (70%)	125 (71%)
Psychologists	62 (65%)	47 (59%)	109 (62%)
National Insurance Institute of Israel	19 (20%)	30 (38%)	49 (28%)
Rehabilitation Department, Ministry of Defense	17 (18%)	23 (29%)	40 (23%)
Educational system	21 (22%)	3 (4%)	24 (14%)
Lawyers or court	19 (20%)	5 (6%)	24 (14%)
Self-referrals	20 (21%)	9 (11%)	29 (17%)
Other	18 (19%)	8 (10%)	26 (15%)
Which percent of the NPA that you conduct is HP-funded?	*N* = 100	*N* = 90	*N* = 190
0%	74 (74%)	50 (56%)	124 (65%)
20%	8 (8%)	4 (4%)	12 (6%)
50%	8 (8%)	5 (6%)	13 (7%)
75%	7 (7%)	9 (10%)	16 (9%)
100%	3 (3%)	22 (24%)	25 (13%)

Note: *HP* Health Plan. Numbers in the top four rows reflect the percentage of participants relative to the total number of participants. Numbers on the bottom four questions reflect the percentage of participants relative to the number of participants who answered the relevant question. For the question about referral source, participants could provide more than one answer and therefore the numbers do not add up to 100%

We asked practitioners to specify the type of patients that could benefit from NPA in terms of medical conditions and personal characteristics. Respondents referred to general conditions, such as a disease of the central nervous system (32%) or complaints of cognitive decline (25%). Across participants, there were 32 specific medical conditions (e.g., hydrocephalus, Parkinson’s disease), and in Table 3 we present only conditions specified by at least 20% of respondents. The majority of respondents (79%) cited traumatic brain injury as a condition that requires NPA, whereas less than a third of the sample mentioned all other conditions (e.g., brain tumors, stroke, attention deficit disorder, epilepsy, and dementia). When asked to specify personal characteristics of individuals who could benefit from NPA, most respondents stated that referral depended on the type of condition (67%). In addition, 33% of respondents mentioned changes in cognitive state as a reason for conducting NPA. All other responses referred to personal characteristics that reflected considerations for psychological treatment, such as rehabilitation potential (47%), younger age (34%), feasibility of return to work (21%), and higher motivation (12%). In response to a question about the advantages of NPA, integration appeared as the main advantage of NPA over other cognitive evaluations (mentioned by 61% of respondents), followed by expertise (45%), breadth (33%), depth (24%), and interpretation (8%). Participants listed length of report (48%), cost (41%), turnaround time (37%), relevance to functioning (16%), and reliability (10%), as the main disadvantages of NPA.

**Table 3:** Attitudes toward neuropsychological assessment (NPA)

Question	Response frequency
Which type of patients could benefit from NPA? (list syndromes)	*N* = 237
Traumatic brain injury	188 (79%)
Brain tumors or other cancer	63 (27%)
Stroke	53 (22%)
Attention deficit disorder	52 (22%)
Epilepsy	50 (21%)
Dementia	48 (20%)
What characterizes individuals who could benefit from NPA?	*N* = 129
A relevant disease	86 (67%)
Rehabilitation potential	60 (47%)
Younger age	44 (34%)
Changes in cognitive state	42 (33%)
Feasibility of return to work	27 (21%)
Higher motivation	16 (12%)
What are the advantages of NPA over other cognitive evaluations?	*N* = 155
Integration	95 (61%)
Expertise	70 (45%)
Breadth	51 (33%)
Depth	37 (24%)
Interpretation	13 (8%)
What are the disadvantages of NPA over other cognitive evaluations?	*N* = 130
Report length	63 (48%)
Cost/price	53 (41%)
Turnaround time	48 (37%)
Relevance to functioning	21 (16%)
Reliability	13 (10%)

Note: Numbers reflect the percentage of participants relative to the number of participants who answered the relevant question. Participants could provide more than one answer and therefore the numbers do not add up to 100%

As shown in Table 4, 99% of the practitioners who answered the relevant questions believed that HPs should fund NPA and that HPs currently pay for an insufficient number of NPAs. Only 24% of respondents reported that they knew how to refer patients to NPA through the HPs. A third (33%) of the participants had heard of the two relevant referral codes, but only half of that number (16%) could distinguish between them. Most respondents (65%) reported that they had clients who paid out of pocket for an NPA that a physician recommended, and the majority of respondents (84%) believed that their clients had a hard time receiving HP funding for an NPA.

**Table 4:** Familiarity with referral procedures

Question	N	Response frequency
Should the HP pay for NPA?	194	192 (99%)
In your opinion, is the number of HP referrals to NPA sufficient?	192	2 (1%)
Do you know how to refer patients to NPA through the HP?	193	46 (24%)
Have you ever heard of code L9616 or code L9617?	189	62 (33%)
Do you know the difference between these two codes?	188	31 (16%)
Have your clients paid for an NPA that a physician recommended?	185	121 (65%)
How difficult is it for your clients to receive HP funding for NPA?	143	120 (84%)

Note: *HP* Health Plan, *NPA* Neuropsychological Assessment. Numbers for all questions, except the last one, reflect the percentage of participants who answered yes relative to the number of participants who answered the relevant question. On the last question, participants rated their response on a Likert scale that ranged from 1 (not at all) to 5 (very much). We present the aggregated percentage of responses 3, 4, and 5 relative to all responses

## Discussion

A range of barriers can prevent Israeli patients from receiving NPA through the healthcare system, from system-related reimbursement considerations to physician-related beliefs, satisfaction, and familiarity with NPA [4]. Our results suggest that patterns of neuropsychological practice in Israel may also contribute to under-funding of NPA through the public healthcare system.

We found that most Israeli neuropsychologists (72%) work in public service, as reported before [6], and that 43% work in hospitals that accept HP vouchers for NPA. Nevertheless, the number of practitioners working in public service was slightly larger than the number of practitioners who performed NPA (68% of the sample), so that employment in public service did not guarantee that the practitioner provided HP-funded NPA. In addition, a smaller proportion of board-certified experts reported that they conducted NPA than the proportion of interns who did so. While interns are obliged to perform NPA due to internship requirements, experts can choose to steer away from NPA and focus on therapy alone. The majority of interns reported that they performed no more than 10 NPAs per year, suggesting that they do not develop their assessment skills beyond the official requirements. The result of these professional practices is that Israeli practitioners perform fewer NPAs per year than the number of NPAs that practitioners in other countries perform per month [7–9]. It is difficult to tell whether the finding that Israeli practitioners perform relatively few NPAs is independent of funding considerations. Hence, the lack of funding may prevent neuropsychologists from providing NPA through the healthcare system, due for example to paucity of assessment positions or to low wages. It is also possible that Israeli practitioners prefer to perform few NPAs, and this preference limits the number of HP-funded NPAs.

We believe that one reason for the low numbers of NPAs that practitioners perform in general, as well as the paucity of HP-funded NPAs, lies in the conceptualization of the profession of neuropsychology in Israel. Vakil and Hoofien [6] noted that Israeli practitioners primarily provide services of case management, vocational counseling, and rehabilitation psychotherapy. Indeed, our respondents cited treatment recommendations as the number one purpose for conducting NPA. Although we did not ask which treatment was at stake, it is possible that most NPAs determine eligibility for psychological and vocational rehabilitation. This impression receives support from the fact that respondents cited psychologists as the second-most common source of referrals to NPA, followed by two rehabilitation agencies (the National Insurance Institute of Israel and the Rehabilitation Department of the Ministry Defense), unlike reports of sources of referrals in North America [7, 10]. Moreover, when asked to define the type of personal attributes that would make a patient eligible for NPA, respondents cited many characteristics typical of considerations for psychological treatment, such as rehabilitation potential, younger age, feasibility of return to work, and higher motivation. Therefore, Israeli practitioners see NPA as part of the rehabilitation services that they provide.

The survey further suggested that although physicians are the main source of NPA referrals, as reported elsewhere [7, 9, 10], only 20% of NPAs in Israel address differential diagnosis and only 3% attempt to establish pre-surgical baseline. These results contrast with reports from other countries in which neuropsychologists reported that the determination of diagnosis was the most common reason for NPA [7–11]. In complementary surveys that targeted physicians, most respondents also indicated that they referred patients to NPA primarily in order to establish or confirm a diagnosis, as well as to establish baseline cognitive functioning [12, 13]. In addition, our participants believed that the greatest advantage of NPA was that it provided in-depth integration. We can speculate that this advantage is of lesser priority to physicians who expect to answer a specific medical question, and it may lengthen the evaluation process as well as the final report. Indeed, physicians often express dissatisfaction from the length of reports and from the related slow turnaround [14]. Israeli practitioners shared these sentiments, and cited report length as the top disadvantage of NPA.

Following report length, Israeli neuropsychologists mentioned cost as the second most common disadvantage of NPA. Lengthy evaluations and report writing clearly involve more work, thus increasing the cost of NPA. All respondents thought that the healthcare system should pay for NPA and that HP funding is insufficient. The majority of practitioners reported that their clients paid out of pocket for an NPA that a physician recommended, and that it was difficult for clients to receive HP funding for NPA. Thus, respondents believed that the Israeli healthcare system should provide neuropsychological services as it provides all other medical care. These attitudes are similar to the attitudes that Israeli physicians expressed [4]. Unfortunately, neuropsychologists also resembled physicians in their lack of familiarity with HP referral procedures to NPA. We note that it is difficult to disentangle cause and effect, since the lack of familiarity not only leads to under-referrals but may also be its result.

## Conclusions

The Israeli healthcare system acknowledges the need for NPA, and the majority of Israeli neuropsychologists work in public service. Nevertheless, most NPAs do not receive appropriate funding within the healthcare system. We identified several possible reasons for under-funding, including the conceptualization of the neuropsychological profession and the mismatch between physicians’ needs and neuropsychologists’ views regarding the main purpose of NPA. One direction for change could be to establish more integrated care teams within the Israeli healthcare system, as done elsewhere [15]. Collaboration between physicians and neuropsychologists could lead to more effective medical treatment, to shorter evaluations and reports (since communication is more direct), and to a decrease in NPA costs. Lastly, to increase healthcare funding for NPA, neuropsychologists should help the healthcare system acknowledge the benefits of NPA. NPA has an incremental value beyond other medical services, especially for the diagnosis of persons with mild cognitive impairment or dementia and for the prediction of outcome of individuals with traumatic brain injury [3]. However, evidence that NPA reduces healthcare costs is still quite preliminary. Such evidence could strengthen our recommendations for both practitioners and policy makers to provide greater funding of NPA within the healthcare system in Israel.

## Data Availability

The data collected for the current study are available from the corresponding author on reasonable request.
